# Controlled synthesis of Mo_2_C micron flowers *via* vapor–liquid–solid method as enhanced electrocatalyst for hydrogen evolution reaction

**DOI:** 10.1039/d3ra04813f

**Published:** 2023-09-04

**Authors:** Yuwei Wang, Jian He, Yipeng Zang, Changbao Zhao, Miaomiao Di, Bin Wang

**Affiliations:** a College of Physical Science and Technology, Bohai University Jinzhou 121013 China; b College of Chemistry and Materials Engineering, Bohai University Jinzhou 121013 China wangbinlhx@163.com; c State Key Laboratory of Catalysis, Dalian Institute of Chemical Physics, Chinese Academy of Sciences Dalian 116023 China

## Abstract

Mo_2_C demonstrates excellent performance in catalysis, and it has been found to possess excellent hydrogen evolution reaction (HER) catalytic activity and highly efficient nitrogen fixation. The catalytic activity of Mo_2_C is greatly influenced and restricted by the preparation method. Sintering and carbon deposition, which affect the catalytic activity of Mo_2_C, are inevitable in the traditional vapor–solid–solid (VSS) process. In this study, we report the controllable synthesis of α-Mo_2_C micron flowers by adjusting the growth temperature *via* a vapor–liquid–solid (VLS) process. The density of the Mo_2_C micron flowers is closely related to the concentration of Na_2_MoO_4_ aqueous solution. The as-grown Mo_2_C micron flowers dispersed with Pt are validated to be an enhanced collaborative electrocatalyst for HER against Pt/VSS-Mo_2_C.

## Abstract 2

Mo_2_C demonstrates excellent performance in catalysis, and it has been found to possess excellent hydrogen evolution reaction (HER) catalytic activity and highly efficient nitrogen fixation.

## Graphical abstract

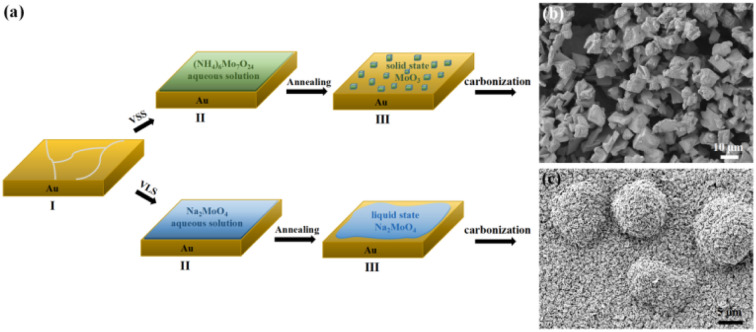

## Introduction

In recent years, transition metal carbides (TMCs) have been intensively researched for their specific chemical properties.^1–3^ TMCs are considered to be similar to precious metals in the aspect of electrochemistry and catalysis. Mo_2_C belonging to the TMC family is called quasi platinum catalyst, and it plays an important role in highly efficient nitrogen fixation^4^ and hydrogen evolution reaction (HER).^5–9^ Especially, as some metal atoms are dispersed on the surface of Mo_2_C crystals for collaborative catalysis, they exhibit excellent selectivity and superior activity for many catalytic reactions.^10–13^ However, many collaborative catalysis suffer from low mass-specific activity owing to the low metal loading.^14^ In order to optimize metal loading, the support Mo_2_C crystals should have a high specific surface area which can provide abundant surface sites to enhance the collaborative catalysis.

As a catalytic material with excellent performance, the catalytic activity of Mo_2_C is greatly influenced and restricted by its preparation method.^15–18^ In earlier studies, the sintering of the as-grown Mo_2_C crystals was inevitable,^19,20^ influencing the structure and morphology of Mo_2_C crystals, which results in the reduction of the specific surface area and catalytic activity. Therefore, it is important to improve the preparation methods to reduce the sintering and thus increase the specific surface area of the Mo_2_C crystals.

Herein, we report the synthesis of α-Mo_2_C crystals *via* an atmospheric pressure vapor–liquid–solid (VLS) method with Na_2_MoO_4_ as the Mo precursor. The morphology of the Mo_2_C crystals could be controlled by adjusting the growth temperature. Mo_2_C micron flowers were obtained when the growth temperature was 780 °C. Compared with the vapor–solid–solid (VSS) mode, VLS mode has the advantages of good wettability and superior mobility, which can promote the lateral migration of Mo precursors and prevent the reactive materials from accumulating.^11–25^ Thus, the as-grown Mo_2_C crystals can form sheet morphology at an appropriate temperature comparing with the block morphology formation at higher temperatures or *via* the VSS mode. The advantage of VLS over VSS mode can be further demonstrated by comparing the HER catalytic activity of the as-grown Mo_2_C dispersed with Pt. Pt/VLS-Mo_2_C has a lower overpotential than Pt/VSS-Mo_2_C at a current density of 10 mA cm^−2^. Mo_2_C crystals grown using the VLS method is of great significance to improve their catalytic activity and expand their application fields.

## Results and discussion

**Fig. 1:**
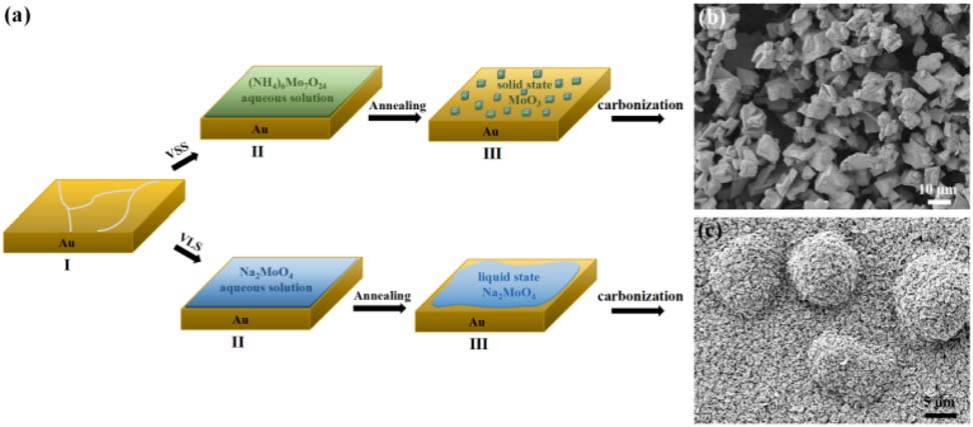
(a) Schematic illustration of the VSS and VLS growth process of Mo_2_C. (b) and (c) Typical SEM images of the Mo_2_C crystals grown with VSS and VLS mode, respectively.

The CVD growth process of Mo_2_C on Au substrate is illustrated in Fig. 1a. The upper panel shows a typical VSS mode for the growth where (NH_4_)_6_Mo_7_O_24_ aqueous solution is used as the Mo precursor. As the growth temperature reaches 780 °C, (NH_4_)_6_Mo_7_O_24_ decomposed to form the solid state of MoO_3_ particles, which were then carbonized to produce Mo_2_C when C_2_H_4_ was introduced into the reaction chamber. Fig. 1b shows the SEM image of the as-grown Mo_2_C with 150 mg per mL (NH_4_)_6_Mo_7_O_24_ as the Mo precursor. The Mo_2_C demonstrated block morphology with size inconsistency.

Mo_2_C micron flowers with high specific surface area were synthesized *via* the VLS mode, and the schemes are shown in the bottom panel of Fig. 1a. 150 mg per mL Na_2_MoO_4_ aqueous solution replaces (NH_4_)_6_Mo_7_O_24_ aqueous solution as the Mo precursor. It is worth noting that the melting point of Na_2_MoO_4_ is 687 °C, it melts into liquid state and forms a liquid–solid interface with Au substrate at the growth temperature (780 °C). Importantly, liquid has the advantage of a lower migration barrier, which is more beneficial to the unrestricted diffusion and homogeneous distribution of the precursors on the Au substrate. Thus, uniform Mo_2_C micron sheets can be synthesized *via* the VLS mode. Moreover, the liquid–solid interface is conducive to the lateral growth of Mo_2_C micron sheets. As the size and density increases, the Mo_2_C micron sheets gradually form the Mo_2_C micron flower morphology, as shown in Fig. 1c.

**Fig. 2:**
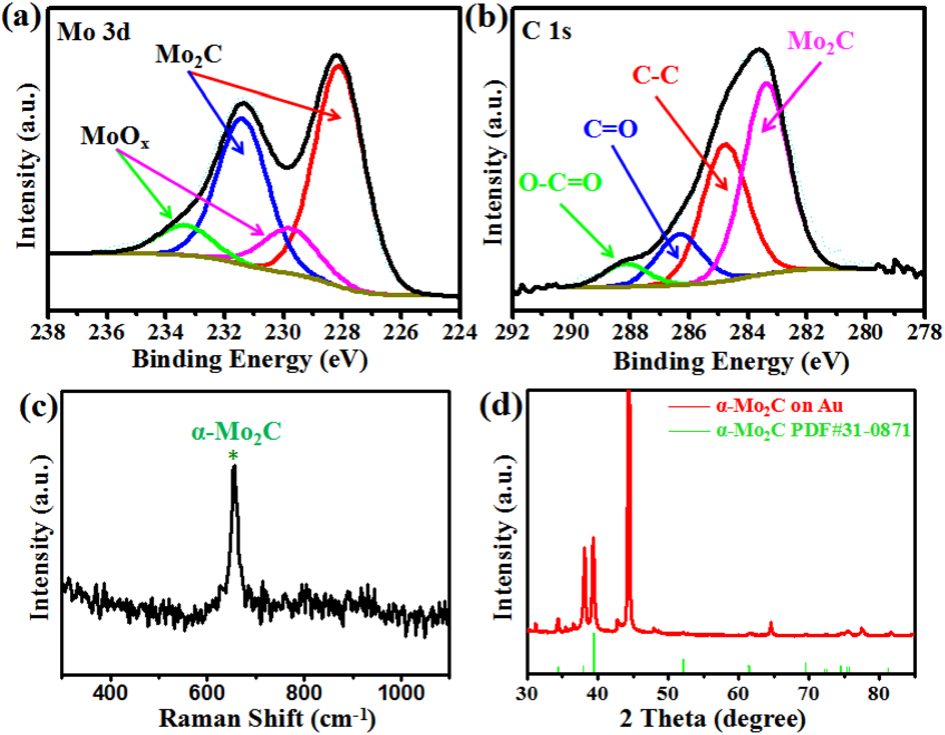
(a) and (b) XPS spectra acquired at the Mo 3d and C 1s regions. Raman spectrum (c) and XRD pattern (d) of the as-grown Mo_2_C nanocrystals.

X-ray photoelectron spectroscopy (XPS) was conducted to evaluate the chemical composition and valence state of the Mo_2_C crystals. Fig. 2a shows the binding energies of Mo 3d peaks at 231.4 eV and 228.1 eV, which are attributed to the Mo 3d_3/2_ and Mo 3d_5/2_, respectively.^26–30^ In addition, two weak peaks were observed at 233.4 and 229.8 eV, representing the intermediate oxidation states of Mo (MoO_*x*_).^28,29^ The MoO_*x*_ may have resulted either from the exposure of Mo_2_C to air or from the oxidization of Mo_2_C during the XPS measurement process. Fig. 2b shows the C 1s XPS spectrum, whereby the peak located at the lower binding energy of 283.3 eV was assigned to C–Mo,^26,27,30^ and those peaks at higher binding energies of 284.8, 286.3, and 288.1 eV can be ascribed to the carbons in the non-oxygenated C–C, C=O, and O–C=O, respectively.^28,29^ The XPS signals confirmed the identity of the Mo_2_C crystals, as expected. Raman spectroscopy and XRD were conducted to evaluate the structure of the Mo_2_C crystals (Fig. 2c and d). Raman spectrum showed a well-defined characteristic peak at 652 cm^−1^, corresponding to the A_g_ mode of α-Mo_2_C crystal.^31,32^ The diffraction peaks of Mo_2_C in the X-ray diffraction (XRD) spectra were consistent with the standard XRD pattern of Mo_2_C (PDF#31-0871), demonstrating that the as-grown Mo_2_C crystals were α-Mo_2_C.

**Fig. 3:**
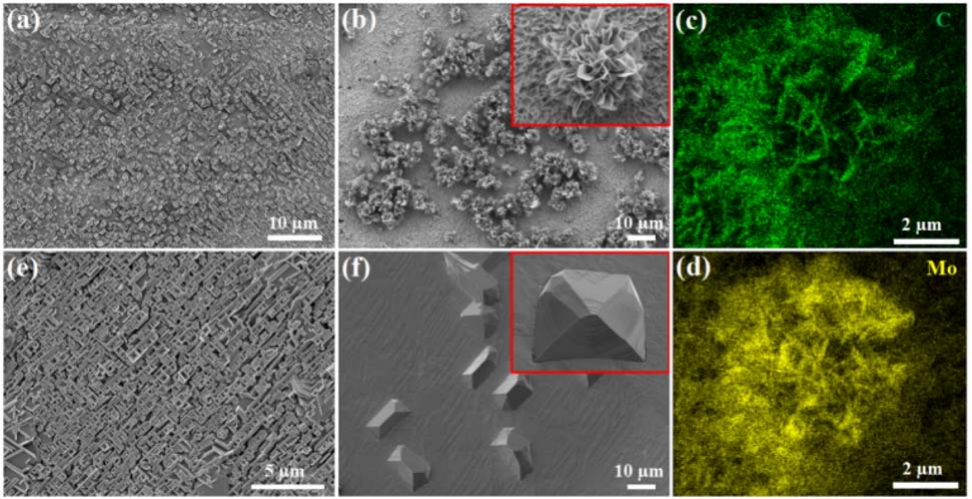
SEM images of the Mo_2_C crystals grown with different concentrations of Na_2_MoO_4_ aqueous solution: (a) 30 mg mL^−1^ and (b) 75 mg mL^−1^. (c) and (d) EDS elemental mapping of Mo and C of the Mo_2_C micron flower, respectively. SEM images of the Mo_2_C crystals grown with different temperatures: (e) 850 °C and (f) 900 °C. Insets in (b) and (f) are SEM images of the individual Mo_2_C crystal grown at 780 and 900 °C with high magnification, respectively.

The morphology and density of Mo_2_C crystals can be tuned remarkably by changing the growth temperature and the concentration of Na_2_MoO_4_ aqueous solution. Fig. 3a and b present the SEM images of the Mo_2_C micron sheets grown with 30 and 75 mg per mL Na_2_MoO_4_ aqueous solutions at 780 °C, respectively. When the Na_2_MoO_4_ aqueous concentration was 30 mg mL^−1^, it provided a low concentration of Mo species, resulting in few nucleation sites, and thus, only a low quantity of Mo_2_C micron sheets appeared, as shown in Fig. 3a. By increasing the Na_2_MoO_4_ aqueous concentration to 75 mg mL^−1^, the shape of the Mo_2_C micron sheets became more evident, whereby some micron sheets have begun to form flower-like shapes. The inset in Fig. 3b is the SEM image of an individual Mo_2_C micron flower. Energy dispersive X-ray spectroscopy (EDS) mapping were recorded for the spatial distribution of the Mo and C elements (Fig. 3c and d), and both of them were found to be distributed uniformly in the micron flowers with sharp edges, exhibiting the uniformity of the Mo_2_C crystals. Subsequently, the influence of growth temperature was investigated, and the 75 mg per mL Na_2_MoO_4_ aqueous solution was used as the Mo precursor. As the growth temperature increased from 850 to 900 °C, the as-grown Mo_2_C crystals gradually adopt block morphology, as shown in Fig. 3e and f. The inset in Fig. 3f is the SEM image of an individual block Mo_2_C crystal.

In order to further demonstrate the advantage of VLS in synthesizing Mo_2_C, the HER catalytic activities of VLS-Mo_2_C and VSS-Mo_2_C were compared. The samples of VLS-Mo_2_C (150 mg per mL Na_2_MoO_4_) and VSS-Mo_2_C [150 mg per mL (NH_4_)_6_Mo_7_O_24_] were synthesized on Au substrates, and both the two kinds of Mo_2_C were loaded with 2 nm Pt for the electrochemical test. The HER catalysis was evaluated in 1.0 M KOH solution using a typical three-electrode system with the studied materials as the working electrodes, Hg/HgO as the reference electrode, and the Pt foil as the counter electrode.

**Fig. 4:**
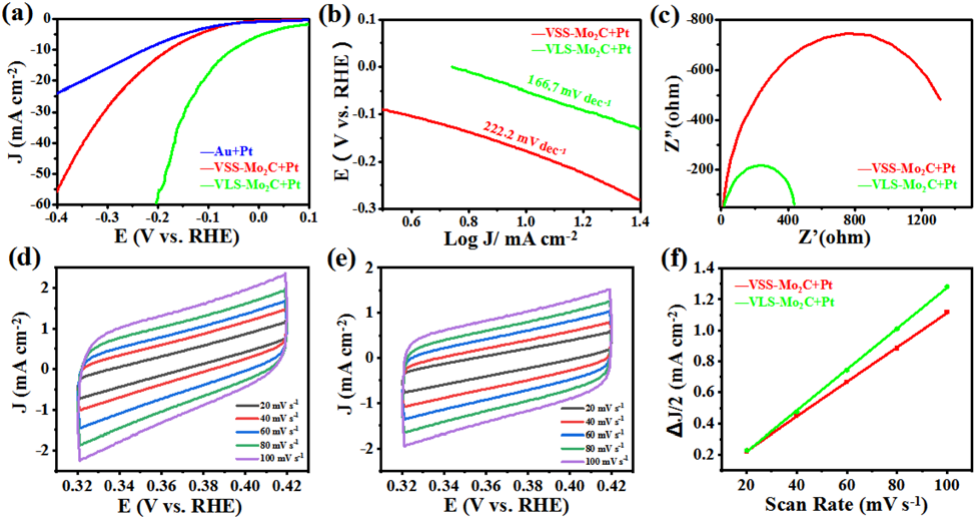
(a) The LSV curves of Pt/VLS-Mo_2_C, Pt/VSS-Mo_2_C, and Pt/Au with IR correction. (b) The corresponding Tafel slopes. (c) Nyquist plots of Pt/VLS-Mo_2_C and Pt/VSS-Mo_2_C collected at the open-circuit voltage. CV curves at different scan rates from 20 to 100 mV s^−1^ of (d) Pt/VLS-Mo_2_C and (e) Pt/VSS-Mo_2_C. (f) The plots of Δ*J versus* scan rates for the Pt/VLS-Mo_2_C and Pt/VSS-Mo_2_C, respectively.

Fig. 4a shows the linear sweep voltammetry (LSV) curves of Pt/VLS-Mo_2_C, Pt/VSS-Mo_2_C, and Pt/Au with a scan rate of 5 mV s^−1^. Compared with the Pt/VSS-Mo_2_C and Pt/Au, the Pt/VLS-Mo_2_C has a lower overpotential of 52 mV *versus* the reversible hydrogen electrode (RHE) at a current density of 10 mA cm^−2^, indicating that the VLS mode can substantially improve the collaborative catalytic performance of Pt/Mo_2_C toward HER in alkaline condition. The derived Tafel slope of Pt/VLS-Mo_2_C and Pt/VSS-Mo_2_C is around 166 and 222 mV dec^−1^, respectively (Fig. 4b), indicating that the hydrogen evolution on both of them undergoes the Volmer mechanism, and water dissociation is the rate-determining step. Critically, a substantially decreased Tafel slope of Pt/VLS-Mo_2_C revealed that the sluggish water dissociation behavior had improved significantly. In addition, electrochemical impedance spectroscopy (Fig. 4c) showed that Pt/VLS-Mo_2_C possessed a lower charge transfer resistance than Pt/VSS-Mo_2_C. The significantly reduced impedance further suggest that Pt/VLS-Mo_2_C can substantially boost the interfacial electron-transfer kinetics between the Mo_2_C and Au foil, which promotes the HER dynamic process. The electrochemical surface areas of Pt/Mo_2_C crystals were further estimated by deriving the electrochemical double layer capacitance (*C*_dl_) from the cyclic voltammetry studies, as shown in Fig. 4d–f. The Pt/VLS-Mo_2_C was found to have a larger *C*_dl_ of 13.2 mF cm^−2^ than Pt/VSS-Mo_2_C (11.1 mF cm^−2^), indicating that the VLS mode can increase the electrochemical surface areas of the as-grown Mo_2_C crystals.

We compared the Pt/VLS-Mo_2_C over the state-of-the-art of electrocatalysts for HER, as shown in Table 1. We believe that the VLS method could offer new insights into the synthetic approaches for Mo_2_C and provide new strategies for constructing metal-loading catalysts with high HER catalytic activity.

**Table 1:** Comparison of some molybdenum carbide based electrocatalysts for HER

Samples	*η*_10_ (mV)	Electrolyte	References
Pt/VLS-Mo_2_C	52	1 M KOH	This work
Mo_2_C@NC/Mo_2_C-12	45	1 M KOH	9
C–MoC-0.5	100	1 M KOH	8
Ni/β-Mo_2_C	157	1 M KOH	33
Co–Mo_2_C@NCNT	186	1 M KOH	34
MoC–Mo_2_C-790	98.2	1 M KOH	35
Mo_2_–MoP NPC/CFP-80	146	1 M KOH	36

## Conclusion

In summary, we demonstrated the VLS growth of α-Mo_2_C micron flowers, which were realized by using liquid precursor for the first time. The morphology and density of the Mo_2_C crystals could be controlled by tuning the growth temperature and concentration of Na_2_MoO_4_ aqueous solution. The unique flower-like structure produces a high specific surface area and abundant surface sites on the surface, increasing the Pt loading and enhancing the collaborative catalysis. The comparison between Pt/VLS-Mo_2_C and Pt/VSS-Mo_2_C in terms of HER catalytic activities further demonstrated the advantage of VLS in synthesizing Mo_2_C crystals. Our study not only offers new insights into the synthetic approaches for Mo_2_C but also provides a new strategy for constructing metal-loading catalysts with high catalytic activity.

## Author contributions

Bin Wang designed and conducted the VLS growth and analyzed the data. Yipeng Zang performed the HER of the materials. All the authors discussed and commented on the manuscript.

## Conflicts of interest

The authors declare that they have no known competing financial interests or personal relationships that could have appeared to influence the work reported in this paper.

## Supplementary Material
